# Efficacy analysis of axillary approach in the treatment of Ideberg type I and II scapular glenoid fractures: Case series

**DOI:** 10.1097/MD.0000000000034333

**Published:** 2023-07-14

**Authors:** Hao Ding, Yong-Gang Bao, Bo Yin, Qing-Hua Chang, Qing-Shu Zai, Qiang Shi, Hua-Jian Hu, Hai-Bin Wang, Yi-Feng Zhao, Fu-Qiang Song, Bin Wu

**Affiliations:** a Department of Clinical Medicine, Jining Medical University, Jining City, China; b Department of Traumatology and Orthopedics, Zoucheng People’s Hospital, Jining City, China; c Department of Traumatology and Orthopedics, Jiaxiang County People’s Hospital, Jining City, China; d Department of Trauma and Orthopedics, Jining Third People’s Hospital, Jining City, China; e Department of Orthopedics, People’s Hospital of Juan Cheng County, Heze City, China; f Department of Orthopedics, Affiliated Hospital of Jining Medical University, Jining City, China.

**Keywords:** axillary approach, fracture fixation, operative technique, scapular glenoid fractures

## Abstract

To investigate the clinical efficacy of the axillary approach in the surgical treatment of Ideberg type I and II scapular glenoid fractures.

**Patient concerns and diagnosis:**

Retrospective analysis of 13 cases of scapular glenoid fracture treated in the affiliated Hospital of Jining Medical College, Jiaxiang County People hospital, Zoucheng City people Hospital, Yanzhou District People Hospital, and Juancheng County people Hospital from December 2020 to January 2022. Eight males (including 1 bilateral) and 5 females, with an average age of 57.5 years (range from 33 to 75 years). According to Ideberg classification, there were 10 cases of type I a, 1 case of type I a combined with type I b, and 2 cases of type II. All patients were treated with axillary approach surgery and 7 patients with combined anterior shoulder dislocation were treated by first-stage manipulation and second-stage reoperation. Seven patients were fixed with a wire anchor, 3 patients with type I a were fixed with a “T” plate, and 5 patients were complicated with rotator cuff tear and were repaired with a wire anchor. At the last follow-up, the Constant-Murley shoulder function score, visual analog score, DASH score, and Hawkins grade were used to evaluate shoulder function, pain, and stability after treatment.

**Intervention:**

The intervention was to treat patients with Ideberg type I and II scaphoid fractures using an axillary approach.

**Outcomes:**

All 13 patients in this group were followed up thoroughly, and the follow-up time was 12 to 25 months, with an average of 18.6 months. The operation time was 65 to 135 minutes, with an average of 85.6 minutes. Intraoperative blood loss ranged from 20 to 120 mL, averaging 55.6 mL. The duration of hospitalization ranged from 7 to 22 days, with an average of 9.6 days. The surgical incisions of all patients were grade-A healing. Bone healing of glenoid fractures was observed 3 months after the operation.

**Lessons:**

The axillary approach for Ideberg type I and II scapular glenoid fractures is a feasible surgical approach with complete access through the muscle gap, minimal surgical trauma, mild postoperative pain, and satisfactory clinical results.

## 1. Introduction

Scapular glenoid fractures are relatively rare clinically. They are often caused by the impact of the humeral head on the scapular glenoid due to high-energy violent shoulder joint injury, accounting for about 10% of scapular fractures.^[1]^ According to the fracture line direction and fracture complexity, Ideberg divides the glenoid fracture into 5 types.^[2]^ Goss supplemented the severe comminuted glenoid fracture to type VI.^[3]^ Early surgical treatment should be performed clinically to improve the function of the shoulder joint.^[4]^ Currently, the standard surgical approaches are the anterior pectoralis major-deltoid and posterior Judet approach. Due to the distribution of many muscles, nerves, and blood vessels around the scapular glenoid, the above surgical approaches for anterior and inferior scapular glenoid fractures have shortcomings, such as insufficient exposure of the operative field, significant trauma, and limited screw fixation direction.^[5]^ Arthroscopic surgery is difficult to fix the fracture with a large volume and fracture line beyond the articular capsule.^[6]^ Professor Tian found that axillary approach surgery for the anterolateral rim of scapular glenoid fractures (mainly Ideberg type Ia) has the advantages of minimal trauma, broad exposure, reliable internal fixation, and concealed appearance.^[7]^ From December 2020 to January 2022, we used the axillary approach to treat 13 patients with scapular glenoid fractures with satisfactory results. This study discussed the curative effect and matters needing attention of this operation through retrospective analysis of the data of this group of patients, as reported as follows.

## 2. Methods and materials

### 2.1. Inclusion and exclusion criteria

#### 2.1.1. Inclusion criteria.

The type of scapular glenoid fracture is Ideberg type I and II; fracture displacement ≥ 4 mm; Fracture involving more than 20% of the shoulder joint surface; patients treated by axillary approach; follow-up observation of fracture healing and shoulder joint function.

#### 2.1.2. Exclusion criteria.

Patients with open fracture; previous history of shoulder injury; patients with severe underlying diseases who could not tolerate surgery.

### 2.2. General data

From December 2020 to January 2022, 13 scapular glenoid fractures were treated surgically with an axillary approach. Eight of them were male (one of them was a bilateral type Ia fracture) and 5 were female, aged 33 to 75 years (mean 57.5 years). The causes of injury: traffic injury in 2 cases, high fall injury in 8 cases, and strain injury in 3 cases. The Ideberg fracture type: 10 cases of type Ia, 1 case of type Ia combined with type Ib, and 2 cases of type II. Combined injuries: ipsilateral greater tuberosity and rotator cuff injury in 5 cases, anterior shoulder dislocation in 7 cases, cranial injury in 2 cases, distal radius fracture in 2 cases, and thoracic injury in 1 case. All patients underwent anterior and lateral scapular X-ray and 3D CT scan before the operation.

### 2.3. Surgical methods

All patients in this study were placed under general anesthesia and operated on in the supine position. The affected upper extremity abducted approximately 90 degrees. The affected limb was sterilized to maintain traction and change position during the operation.

The incision extends from the top of the axilla along the anterior border of the latissimus dorsi muscle to the middle level of the outer border of the scapula (approximately 7 cm). The skin, subcutaneous tissue, and deep fascia are incised along the anterior border of the latissimus dorsi muscle and bluntly separated. The humeral head can be palpated on the cephalad side of the incision. The axillary nerve and the posterior rotator humeral artery can be traced by the finger distal to the articular surface of the humeral head, which is protected and does not need to be free. The lateral border of the scapula is found inward along the anterior border of the latissimus dorsi muscle, and the lateral border of the scapula is bluntly separated forward and backward, respectively. Hohmann hooks are then inserted anteriorly and posteriorly, pulling the muscles apart. Then we can see that the Subscapular artery is located in the front, and the spiral scapular artery is located below. In the quadrilateral area surrounded by the above vessels and nerves, the glenohumeral joint capsule, the lateral edge of the scapula, and the inferior and anterior part of the scapular glenoid can be clearly exposed. Depending on the fracture, the decision is made whether to incise the joint capsule, traction the affected limb and increase the joint space.

Most of the scapular glenoid fracture fragments were easily reduced. Then the reduction was maintained by using reset forceps, followed by driving a Kirschner wire guide in the direction of the vertical fracture line. Finally, a small plate or herbert nail was chosen according to the size of the fracture fragment. In this group miniature “T” plates were used in 2 patients with type Ia. The remaining eleven glenoid fractures were fixed with herbert nails and suture anchors. In 5 patients with combined greater tuberosity fractures and rotator cuff laceration, after fixing the scaphoid fracture, a longitudinal incision was made lateral to the shoulder joint to expose and reduce the greater tuberosity fracture A 5.0 mm suture anchor was placed in the proximal humerus to reconstruct the rotator cuff. We confirmed that the scapular glenoid fracture was well-reducted and fixed during the surgery by intraoperative front and side x-rays of the scapula. Then, the surgical area was flushed, and a drainage tube was placed depending on the blood leakage, and finally, the incision was closed layer by layer.

### 2.4. Postoperative management

The patient was given antibiotics once postoperatively, and the affected limb was suspended using a triangular sling. Patients with intraoperative drains were removed within 48 hours postoperatively. In this study, 5 patients with rotator cuff lacerations had the affected limb immobilized with an abduction brace for 4 weeks, followed by functional exercise. The remaining patients underwent passive functional exercises of the affected shoulder joint starting on the second postoperative day, with one set each day in the morning and evening. The exercise intensity was based on the patient’s feeling of mild pain. Partial weight-bearing and resistance exercises were started after 4 weeks, and full weight-bearing and greater-strength resistance exercises were started after 8 weeks. Regular postoperative follow-up X-rays evaluated the efficacy at 1, 2, 3, and 5 months.

## 3. Result

All 13 patients in this group were followed up thoroughly, and the follow-up time was 12 to 25 months, with an average of 18.6 months. The operation time was 65 to 135 minutes, with an average of 85.6 minutes. Intraoperative blood loss ranged from 20 to 120 mL, averaging 55.6 mL. The duration of hospitalization ranged from 7 to 22 days, with an average of 9.6 days. The surgical incisions of all patients were grade-A healing. Bone healing of glenoid fractures was observed 3 months after the operation. At the last follow-up, the shoulder range of motion was forward flexion 160.5°±9.3°, abduction 155.6°±12.5°, external rotation (neutral position) 40.7°±5.8°, internal rotation (neutral position) 65.4°±12.1°. The Constant-Murley score for the shoulder joint was (92.3 ± 6.5) points (range 85–95), and the DASH score was (12.16 ± 6.86) points (range 3.26–26.75). The Hawkins scale was used at the last follow-up to evaluate glenohumeral stability. All 13 patients were grade A. None of the patients had incision infection, vascular and nerve injury, or internal fixation loosening or rupture.

## 4. Discussion

### 4.1. Anatomical characteristics and technical points of the axillary approach

The anatomy of the axilla is very complex. The axillary artery and the brachial plexus nerve cross the axilla. However, in the middle of this complex anatomy, there is a natural gap between the latissimus dorsi and subscapularis muscles. The operator can reach directly below and in front of the scapular glenoid, which is the anatomical basis of the axillary approach.^[8]^ Because of the dense sweat glands in the center of the axilla, we divided the axilla into 3 equal parts from anterior to posterior and chose the middle and posterior 1/3 as the incision for the axillary approach, close to the anterior border of the latissimus dorsi muscle and entered between the latissimus dorsi and subscapularis muscles. The essential structures, such as the brachial plexus nerve and axillary artery, are in front of the subscapularis muscle. The relevant tissues can be pushed away along the front of the latissimus dorsi muscle. The humeral head can be palpated by abducting the affected limb at about 90°, and the axillary nerve and the posterior rotator humeral artery can be palpated on the proximal part of the humeral head which is protected with care. A blunt separation of the tissue along the scapular glenoid downward will reveal the spiral scapular artery. The subscapular vessels and the thoracodorsal nerve can be revealed in an anterior-inferior direction. The humeral glenoid joint can be fully exposed in the area enclosed by those mentioned above neurovascular. The joint capsule, humeral head, and fracture fragment can be revealed after pulling with 2 Hoffman hooks.

In China, Professor Tian pioneered anatomical studies and concluded that axillary approach surgery is less invasive, with concealed and beautiful incisions, and can achieve good clinical outcomes.^[7]^ Among the 13 scapular glenoid fractures in this study, Ideberg type Ia scapular glenoid fractures accounted for a relatively large number of 10 cases. Unlike bony Bankart injuries, most of the Ideberg type Ia scapular glenoid fractures involved in this paper are anterior and inferior to the scapular glenoid.^[9]^ Following fracture displacement, the glenoid labral tissue attached to the fractured fragment is often torn anterior and superior to the scapular glenoid rather than at the fracture site. Therefore, it is only necessary to reveal the inferior anterior part of the scapular as described above, and the reduction and fixation are relatively simple. The shortest incision in this group of patients is only 6 cm.

**Figure 1.**
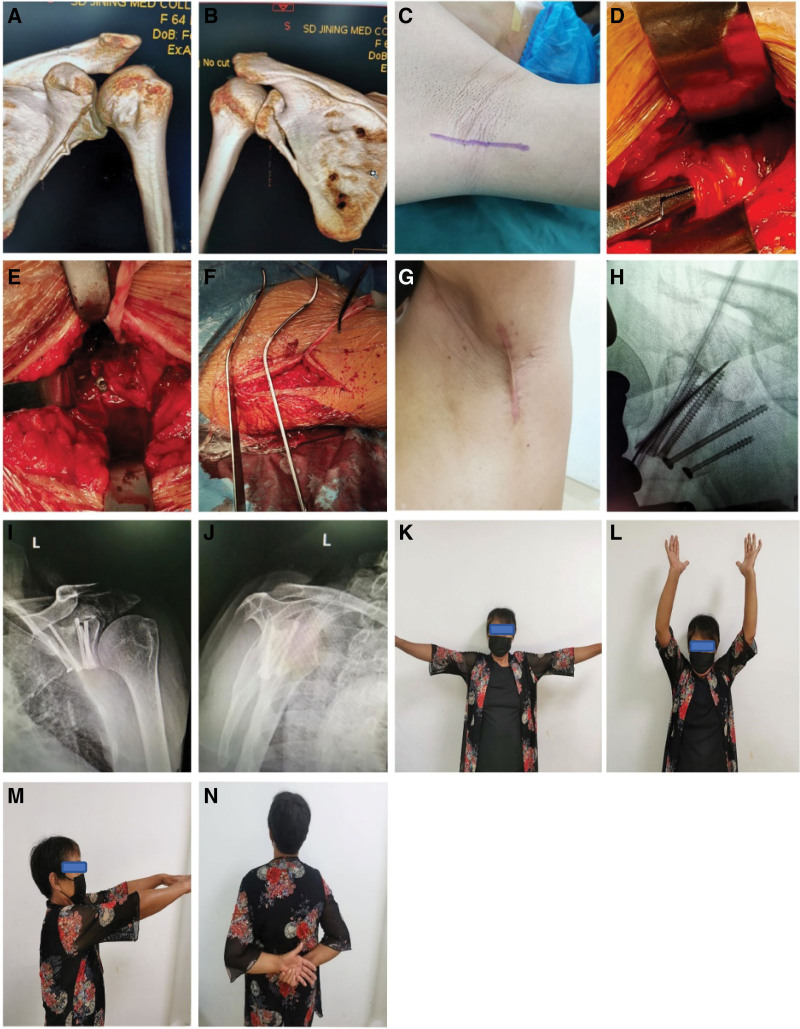
Female patient, 64 years old, was hit by a car while riding her electric bicycle. Preoperative CT 3D reconstruction (A, B) shows an Ideberg II scapular glenoid fracture with the scapular glenoid fracture line extending to the lateral edge of the scapula. The operation was treated by axillary approach with general anesthesia in the supine position, the incision was made from the apex of the axilla along the anterior border of the latissimus dorsi muscle towards the proximal end of the trunk to the level of the subscapular angle (C), and the axillary nerve and the posterior spinohumeral artery were revealed (D). The glenohumeral capsule is further exposed by blunt inward separation along the anterior border of the latissimus dorsi muscle gap, and the fracture is subsequently repositioned and fixed with screws (E). The Hohmann pulling hook was intraoperatively used to pull the muscle (F). Intraoperative X-ray fluoroscopy reveals a well-positioned scaphoid fracture with a hollow screw (H). The incision is closed with sutures, approximately 7 cm long (G), and the incision can be obscured when the affected limb drops. Positive and lateral x-rays of the scapula of the shoulder joint 3 months after surgery showed that the scapular pelvis fracture was well reset (I, J), and the patient’s shoulder function returned to normal (K–N).

For fractures involving the distal extension of the lateral border of the scapula, which requires extended surgical exposure, we chose to dissect the lateral border of the scapula from the distal end of the spiral scapula artery for reduction and fixation intraoperatively, and this group involved 2 such patients. This study has a unique case of a patient with a combined fracture of the scapular anterior inferior and inferior posterior rim. The fracture was first fixed anteriorly, and then it was necessary to extend the surgical exposure posteriorly and fix the fracture from posterior to anterior superiorly (Fig. 1).

### 4.2. Advantages and feasibility of the axillary approach

The most commonly used surgical approaches are anterior and posterior for Ideberg I and II fractures of the scapular glenoid. However, both of these approaches require partial severance of muscle tissue, which is traumatic and slow in postoperative recovery.^[10]^ Due to the deep anatomical location of the scapular glenoid, the currently used open surgical approach only partially reveals the inferior anterior aspect of the scapular glenoid, which increases the difficulty of surgery and inconveniences clinical treatment. The pectoralis major-deltoid approach can also be used for avulsion fractures of the anterior inferior border of the scapular glenoid. However, there are disadvantages to the pectoralis major-deltoid approach, such as limited intraoperative exposure and inability to visualize the scapular neck, difficulty placing the screws perpendicular to the fracture line, and the need to partially sever the subscapularis during the procedure. Even if intraoperative suture repair is performed, it generally impacts the shoulder joint’s stability and range of motion after surgery.^[4]^ The Judet approaches are primarily used for posterior scapular glenoid fractures,^[11]^ which also require partial severance of the posterior deltoid muscle bundle. The Judet approach has disadvantages such as long incisions, trauma, and easy damage to supra-scapular vessels, axillary nerves, and related muscles; the modified Judet approach also has the risk of postoperative flap necrosis and subcutaneous hematoma.^[12]^ Most anterior scapular glenoid fractures occur on the inferior anterior aspect of the scapular glenoid.^[13]^ The axillary approach applied in this study can reveal the anterior, inferior, and lateral margins of the scapular glenoid and even part of the posterior margin, which coincides with the frequent sites of scapular glenoid fractures. The incision in the axillary approach starts from the mid-posterior aspect of the top of the axilla. It travels in the interval from the latissimus dorsi, teres major, and subscapularis muscles. In addition, there is no need to cut any muscle tissue in the axillary approach, which is less traumatic and causes less bleeding. The minimum intraoperative bleeding in our study patients was only 20 mL, with an average of 55.6 mL.

The axillary approach is suitable for Ideberg type I and II scapular glenoid fractures covered in this study. Ideberg type I and II scaphoid fractures can be fixed surgically using nails, suture anchor, microplates, and special plates for the axillary approach to fix the fracture.^[7]^ When we use the axillary approach for surgery, the fractured fragments can be nudged directly into place with the attached joint capsule because the direction of displacement of the broken mass is perpendicular to the direction of the operator’s view. Temporary fixation is achieved by applying a parallel articular surface vertical fracture line with a Kirschner wire. Then the fracture is reduced and fixed by X-ray fluoroscopy in the tangential position of the scapular glenoid. Subsequently, hollow nails, anchor nails, and “plates” are placed to achieve compression fixation of the fracture. All 10 patients with Ideberg type Ia fractures in this study were fixed with rigid internal fixation using herbert nails, including 5 patients with anchor nails and suture fixation of the fracture fragment to the glenoid labrum and 2 patients with microplates. The 2 patients with Ideberg type II scaphoid glenoid fractures in this study had large fracture fragments that would have required extensive tissue separation and would have been more traumatic if plates had been placed. Ultimately 2 we chose to use multiple herbert nails for rigid internal fixation. In this study, 1 patient with Ideberg type Ia combined with Ideberg type Ib was fixed with herbert nails and anchor nails. All patients had secure intraoperative fracture fixation, and early rehabilitation was started immediately after surgery. Axillary approach surgery can be performed in either the supine or lateral position.^[7]^ All patients in this study were placed in the supine position, which means that the affected shoulder is elevated to facilitate the operator’s recognition of the axillary anatomy during surgery. And this is also a choice based on the operator’s familiarity with the position and surgical habits and facilitates anesthetic management of the patient and fluoroscopic operation during surgery.

### 4.3. Operation precautions and postoperative rehabilitation

There is a rich distribution of blood vessels and nerves in the axilla, so the surgeon should master the anatomical structure of the axilla and be gentle when separating the blood vessels and nerves. The correct location of the incision is the critical point of the surgery. The best location for the incision is at the anterior border of the latissimus dorsi muscle in the middle and posterior 1/3 of the axilla. In contrast, the anterior incision easily separates the axillary vessels and the brachial plexus nerve, and the posterior incision is challenging to pull the latissimus dorsi muscle. Careful intraoperative identification of the glenohumeral space is another critical element of the procedure. Due to the instability of the shoulder joint, muscle relaxation after anesthesia can cause the shoulder joint to be in a dislocated position, especially in cases of combined preoperative dislocation. Wang et al^[14]^ reported 12 scapular fractures, 2 of whom had a combined shoulder dislocation and 3 patients with a significant tuberosity fracture. All of these had shoulder instability and were highly susceptible to dislocation, especially after anesthesia. In this study, 7 patients with shoulder dislocations occurred during surgery, and we first performed manual pushing of the humeral head to reduce the shoulder joint. After reduction, we could observe the fracture site of the scapular glenoid. The scapular glenoid articular surface has an inward inclination of approximately 30° to the sagittal plane, so attention should be paid to the angle of the screw when placing the nail, and it is best to reveal the glenohumeral joint space. If the fractured fragment at the anterior border of the scapular glenoid is large, intraoperative fixation can be performed with a buttress plate. However, in patients with Ideberg type III, IV, and V fractures involving the superior scapular glenoid fracture, the axillary approach does not reveal the fracture mentioned above and, therefore, cannot be used. Compared with other parts of the incision, the sweat glands are well-developed in the axilla. It is challenging to keep the incision dry after surgery, which increases the risk of infection and poor incision healing. The affected limb should be abducted as much as possible to keep the incision dry and clean. As the postoperative period progresses, the contracture of the axillary incision scar may affect the abduction function of the shoulder joint of the affected limb.

Studies have shown that for scapular fractures, early initiation of functional shoulder exercises is advocated in patients with good postoperative fracture stability.^[4,15]^ When the axillary approach was used, no muscles around the shoulder joint were cut during the operation. Only the lower shoulder capsule was partially cut to rebuild the stability of the shoulder joint, all of which achieved the requirement of early functional exercise. In this study, except for 5 patients with combined greater tubercle fracture and rotator cuff injury repair and reconstruction, all patients started the active and passive functional exercise of the affected shoulder joint mobility on the second postoperative day under the guidance of the physiatrist. All patients in this study had good shoulder mobility at the end of the follow-up period, and no shoulder instability was observed.

### 4.4. Limitations of this study

Scapular glenoid fracture is relatively rare in the clinic. This study was a multicenter retrospective study, but the number of cases is small, and the follow-up time is short. Further prospective, large sample size, and long-term case-control studies are needed to demonstrate this operation’s long-term clinical effects and potential late complications.

## 5. Conclusions

In summary, the axillary approach for the treatment of Ideberg type I and type II glenoid fractures has less surgical trauma, less bleeding, a clear surgical field, and good postoperative shoulder joint function recovery. It is a beautiful and practical surgical approach for treating Ideberg type I and type II glenoid fractures.

## Acknowledgments

The authors would like to thank our department colleagues and these patients for their dedication, and those patients had signed the informed consent forms.

## Author contributions

**Conceptualization:** Bin Wu.

**Data curation:** Bo Yin, Qing-Hua Chang.

**Formal analysis:** Hao Ding, Qing-Shu Zai.

**Investigation:** Qiang Shi, Fu-Qiang Song.

**Project administration:** Hao Ding, Bin Wu.

**Software:** Hai-Bin Wang, Hua-Jian Hu.

**Supervision:** Bin Wu, Yi-Feng Zhao.

**Writing – original draft:** Hao Ding, Yong-Gang Bao.

**Writing – review & editing:** Bin Wu.
